# Repeatability, Reproducibility and Standardisation of a Laser Doppler Imaging Technique for the Evaluation of Normal Mouse Hindlimb Perfusion

**DOI:** 10.3390/s130100500

**Published:** 2012-12-28

**Authors:** Adelaide Greco, Monica Ragucci, Raffaele Liuzzi, Sara Gargiulo, Matteo Gramanzini, Anna Rita Daniela Coda, Sandra Albanese, Marcello Mancini, Marco Salvatore, Arturo Brunetti

**Affiliations:** 1 Dipartimento di Scienze Biomediche Avanzate, University Federico II, Via Pansini 5, 80137 Naples, Italy; E-Mails: sandralbanese@gmail.com (S.A.); marsalva@unina.it (M.S.); brunetti@unina.it (A.B.); 2 CEINGE, Biotecnologie Avanzate,Via Gaetano Salvatore 482, 80145 Naples, Italy; 3 Fondazione IRCCS SDN, Instituto per la Ricerca e l'alta Formazione in Diagnostica Nucleare, Via Gianturco 113, 80143 Naples, Italy; E-Mail: monique.13@hotmail.it; 4 Istituto di Biostrutture e Bioimmagini, Consiglio Nazionale delle Ricerche, Via Tommaso De Amicis 95, 80145 Naples, Italy; E-Mails: raffaele.liuzzi@cnr.it (R.L.); sara.gargiulo@ibb.cnr.it (S.G.); matteo.gramanzini@ibb.cnr.it (M.G.); danielacoda86@gmail.com (A.R.D.C.); marcello.mancini58@gmail.com (M.M.)

**Keywords:** Laser Doppler Perfusion Imaging, hindlimb perfusion, mice, microcirculation, standardisation

## Abstract

**Background:**

Preclinical perfusion studies are useful for the improvement of diagnosis and therapy in dermatologic, cardiovascular and rheumatic human diseases. The Laser Doppler Perfusion Imaging (LDPI) technique has been used to evaluate superficial alterations of the skin microcirculation in surgically induced murine hindlimb ischemia. We assessed the reproducibility and the accuracy of LDPI acquisitions and identified several critical factors that could affect LDPI measurements in mice.

**Methods:**

Twenty mice were analysed. Statistical standardisation and a repeatability and reproducibility analysis were performed on mouse perfusion signals with respect to differences in body temperature, the presence or absence of hair, the type of anaesthesia used for LDPI measurements and the position of the mouse body.

**Results:**

We found excellent correlations among measurements made by the same operator (*i.e*., repeatability) under the same experimental conditions and by two different operators (*i.e*., reproducibility). A Bland-Altman analysis showed the absence of bias in repeatability (p = 0.29) or reproducibility (p = 0.89). The limits of agreement for repeatability were –0.357 and –0.033, and for reproducibility, they were –0.270 and 0.238. Significant differences in perfusion values were observed in different experimental groups.

**Conclusions:**

Different experimental conditions must be considered as a starting point for the evaluation of new drugs and strategic therapies.

## 1. Introduction

The mouse is the most common animal model for the study of the pathogenesis of human diseases and for testing new therapies [1]. Over the past decade, innovative technologies and the miniaturisation of biomedical devices have been developed to obtain quantitative, accurate and reproducible information in rats and mice [2,3].

Laser Doppler is one of the imaging techniques used in biomedical research. This technique causes minimal pain and distress to mice, reduces the number of animals necessary in biomedical protocols, and allows chronic, non-terminal assessment of physiological superficial perfusion in mice. Laser Doppler measures the total local microcirculatory blood perfusion including the perfusion in capillaries (*i.e*., nutritive flow), arterioles, venules and shunting vessels. This technique is commonly used to monitor the effect of environmental conditions, physical manipulations, and drug treatments on tissue perfusion. Laser Doppler detects shifts in the frequency of laser light (*i.e*., Doppler shift) after it interacts with moving components of tissues such as red blood cells [4–6]. The measurement depth depends on tissue properties such as the structure and density of the capillary beds, pigmentation and oxygenation. It also depends on the wavelength of the laser light and on the distance between the sending and receiving fibres in the laser Doppler probe. In normal skin, using Perimed laser Doppler instruments, a probe with standard fibre separation (0.25 mm), and a 780 nm wavelength laser, the measurement depth will be approximately 0.5–1 mm. No current laser Doppler instrument can provide absolute perfusion values (e.g., mL/min/100 gram tissue). Rather, measurements are expressed in arbitrary Perfusion Units (PU). To allow the comparison of results, it is absolutely essential to calibrate the laser Doppler.

Fibre Optic Monitors and Laser Doppler Perfusion Imaging (LDPI) are the two types of laser Doppler instruments used to measure blood flow. Laser light is transmitted via a fibre optic probe placed in direct contact with the tissue under investigation. Several fibre optic probes are commercially available. Non-invasive continuous measurements of flux are made with skin surface probes. Needle probes designed to penetrate tissues are used intraoperatively to measure flow within tissues such as the brain or liver. Endoscopic probes are less invasive than needle probes and measure flow to mucosal surfaces. Laser Doppler Perfusion Imaging is a non-invasive system that measures flux within tissues without the use of fibre optic probes. A moving mirror directs reflected laser light from moving red blood cells to a photodetector. Subsequent processing results in a two-dimensional color-coded image of flux [7]. One of the applications of Laser Doppler Perfusion Imaging in biomedical research is the assessment and monitoring of the microcirculation in mouse and rat models of hind limb ischemia for studying peripheral arterial disease (PAD) [8]. This disease is characterised by the presence of partial or complete occlusion of peripheral arteries, especially in the lower limbs. To date, medical therapies for symptomatic relief are limited. Although surgery is useful for some individuals, the long-term results are disappointing. Therefore, there is an urgent need for new therapies for the treatment of peripheral arterial disease. To test new therapies, animal models of hind limb ischemia have been developed. Compared to other models of tissue ischemia, such as coronary or cerebral artery ligation, the occlusion of the femoral artery is a simpler model with easy access to the femoral artery and a low animal mortality. Niiyama *et al.* have demonstrated a simple and reproducible method for the study of peripheral artery disease in a mouse model of hind limb ischemia [8]. In this context, the LDPI technique is useful to evaluate the effectiveness of therapy with angiogenic [9] or antiangiogenic drugs [10,11]. However, there are no current guidelines for LDPI acquisition in mice. Furthermore, how experimental conditions such as body temperature or the type of anaesthesia could influence results is unknown. The goal of our study was to standardise LDPI methodology for routine mouse studies to obtain accurate quantitative data and a normal database of perfusion values in mice.

## 2. Experimental Section

### 2.1. Materials and Methods

#### 2.1.1. Animal Studies

Animal studies were performed in accordance with NIH recommendations and with the approval of the institutional animal research committee. Animal care and the humane use and treatment of mice were in strict compliance with institutional guidelines, the Guide for the Care and Use of Laboratory Animalsand the Association for Assessment and Accreditation of Laboratory Animal Care International [12]. The procedures related to animal use conformed to all regulations protecting animals used for research purposes, including those of the DL 116/92. All imaging studies were performed under general anaesthesia, and a veterinarian evaluated the mice for any signs of distress. We used a total of 48 8-week-old female CD1 mice (Harlan, Animal Research Laboratory, UK) for LDPI standardisation, and 20 of those mice were subjected to another LDPI analysis for repeatability and reproducibility evaluation.

#### 2.1.2. Repeatability and Reproducibility

Twenty 8-week-old female CD1 mice were analysed (Harlan, Animal Research Laboratory, UK). A repeatability and reproducibility analysis was performed on blood perfusion signal data before the standardisation analysis. All of the mice were evaluated under the same experimental conditions. They were placed in dorsal decubitus, anaesthetised using isoflurane (4% induction dose, 2.0% maintenance dose) plus oxygen (1 L/min). The mice were shaved and maintained at a 36 °C body temperature. Concerning reproducibility, two operators (the first operator, MR, had less than five years of experience in molecular imaging and the second operator, AG, had more than five years of experience in imaging) performed the measurements of blood perfusion under the same experimental conditions. The median perfusion values are reported.

#### 2.1.3. Standardisation of LDPI

We assessed the variability of measurements of hindlimb microcirculation under different experimental conditions as follow:

1. Hair of the hind limbs removed with a clipper (ARC) *versus* hair removed with an apposite depilatory cream (ARD);
2. dorsal *versus* ventral decubitus position;
3. inhalation anaesthesia performed using isoflurane (4% induction dose, 2.0% maintenance dose) plus oxygen (1 L/min). Subsequently, mice were placed in the selected decubitus position;
4. injectable anaesthesia. A combination of 100 mg/kg of ketamine plus 10 mg/kg of xylazine was injected intraperitoneally. After LDPI acquisition, 1 mg/kg atipamezole was injected into the mice for anaesthetic reversal;
5. The desired body temperature (36 °C *versus* 38 °C) was achieved through the use of a thermo-heated carpet and an infrared lamp. Temperature was monitored with an endorectal probe.

The 48 mice received 8 LDPI measurements that lasted 5 minutes each, for a total of 45 minutes of anaesthesia per mouse. Perfusion measurements were performed under the same body conditions except for the body temperature. Twenty-four mice were analysed at 36 °C body temperature and the remaining 24 mice at 38 °C.

#### 2.1.4. Laser Doppler Perfusion Imaging

LDPI measurements were performed with the Laser Doppler Perfusion Imager “Periscan PIM II” (PERIMED Ltd., Italy). We used a solid state low power laser with a wavelength of 670 nm. The laser is directed into the tissue by a system of motorised mirrors, and it scans the predetermined area. The laser beam is interrupted for approximately 35 milliseconds before each measurement to avoid motion artefacts. Periscan PIM II is based on the well-known Doppler principle. The Doppler effect, caused by the interaction between the laser and red blood cells, is translated graphically as a coloured dot. The set of scanned points formed a colour map with a chromatic scale from blue to red expressed in Volts. In addition, a picture in black and white based on the total record of the reflected light is provided. For these experiments, we used the “high” resolution in single mode. The sample matrix has a dimension of 64 × 64 points. The acquisition of each image requires the perfect immobilisation of the animal for approximately 5 minutes under general anaesthesia. After the induction of anaesthesia, we allow 10 minutes of equilibration time to ensure a stable body temperature and for the mice to reach a deep plane of anaesthesia as indicated by the absence of the paw reflex. The perfusion values were measured on the obtained image. The measurement consists of manually drawing regions of interest (ROI), extended between the femoral-tibial-patellar joint and the caudal end of the phalanges of each limb. Then, the mean and median of perfusion in each ROI is calculated. The software allows the immediate export of all collected data to a spreadsheet in Microsoft Excel.

#### 2.1.5. Statistical Analysis

The results of continuous variables are reported as medians and interquartile ranges. The statistical significance of differences between groups was estimated using the Mann-Whitney non-parametric unpaired test and *p* values of <0.05 were considered statistically significant. Bland-Altman analysis was used to evaluate repeatability and reproducibility. All statistics in this study were performed using SPSS for Windows (Statistical Package for the Social Sciences; SPSS Inc., Chicago, IL, USA).

## 3. Results and Discussion

### 3.1. Repeatability and Reproducibility

**Figure 1.**
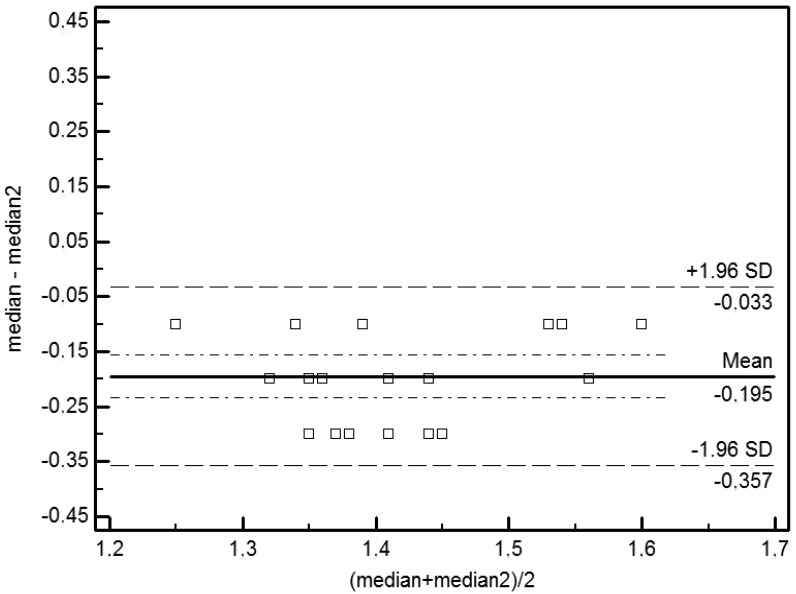
Bland-Altman analysis of the repeatability of LDPI measurements under different experimental conditions. Bias is represented by the solid line (–0.195). The two dotted lines represent the limits of agreement for repeatability (–0.357; –0.033).

**Figure 2.**
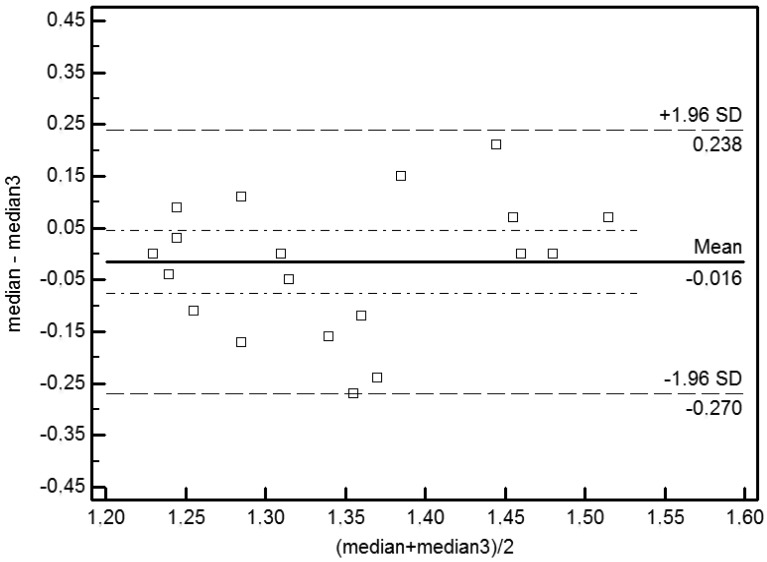
Bland-Altman analysis of the reproducibility of LDPI measurements under different experimental conditions. Bias is represented by the solid line (–0.016). The two dotted lines represent the limits of agreement for reproducibility (–0.270; 0.238).

Bland-Altman (BA) plots were used to detect agreement and bias. The graph shows the average of two measures on the horizontal axis (*i.e*., x-axis) and the difference between two values on the ordinate (*i.e*., y-axis). Bland–Altman (BA) plots showed an absence of bias in measures of repeatability (p = 0.29) and of reproducibility (p = 0.89). The averages observed for the repeatability (–0.195) and reproducibility (–0.016) are not significantly different from 0 (p > 0). The limits of agreement are expressed as averages of the differences ±1.96 SD. The limits of agreement for repeatability are –0.357 and –0.033 and for reproducibility are –0.270 and 0.238 (Figures 1 and 2).

### 3.2. Standardisation

Medians and interquartile ranges are reported in Tables 1 and 2. The Mann-Whitney test was applied to results from experiments in the dorsal decubitus position *versus* the ventral decubitus position (Tables 3 and 4). Only the significant tests are reported.

**Figure 3.**
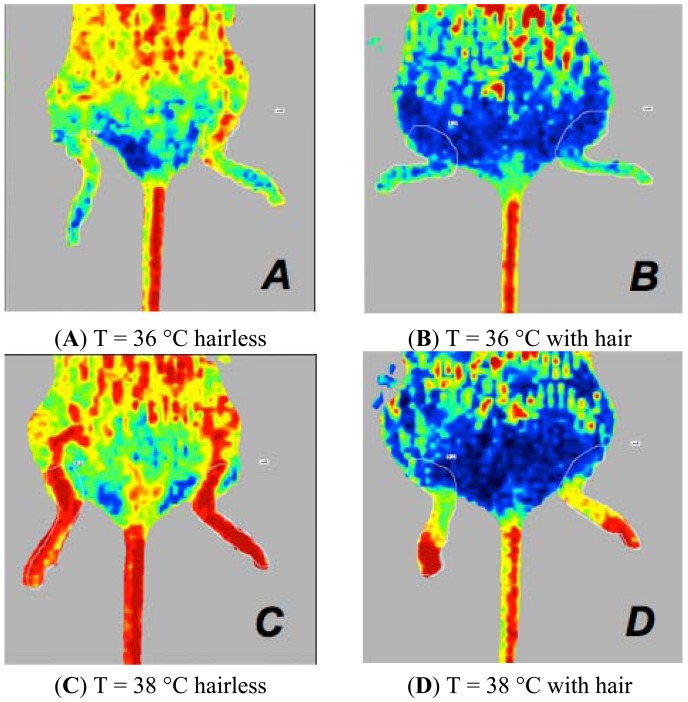
Laser Doppler images of the hind limbs of mice anaesthetised with isoflurane at 36 °C (**A**,**B**) or at 38 °C (**C**,**D**). Perfusion signals in mice depilated with ARC and ARD, placed in the dorsal decubitus position. ARD mice (A,C) show higher perfusion values compared to ARC mice (B,D).

**Figure 4.**
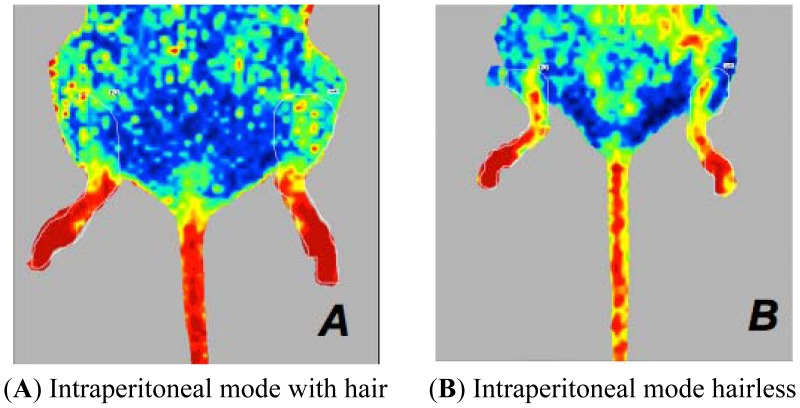

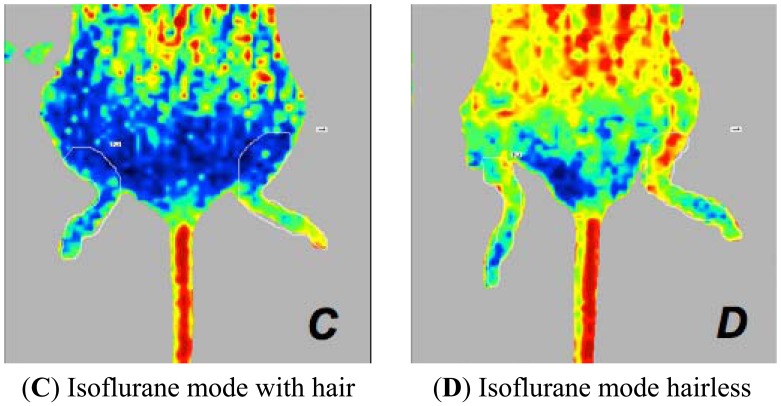
Laser Doppler images of the hind limbs of mice. Perfusion signal is associated with the type of anaesthesia when the mouse is placed in dorsal recumbency. These mice have a body temperature of 36 °C **(A**,**B**,**C**,**D)**. Intraperitoneally anaesthetised mice (A,B) show higher perfusion values than those of the isoflurane-anaesthetised mice (C,D), in comparison to ARC mice (A,C) or ARD mice (B,D).

**Figure 5.**
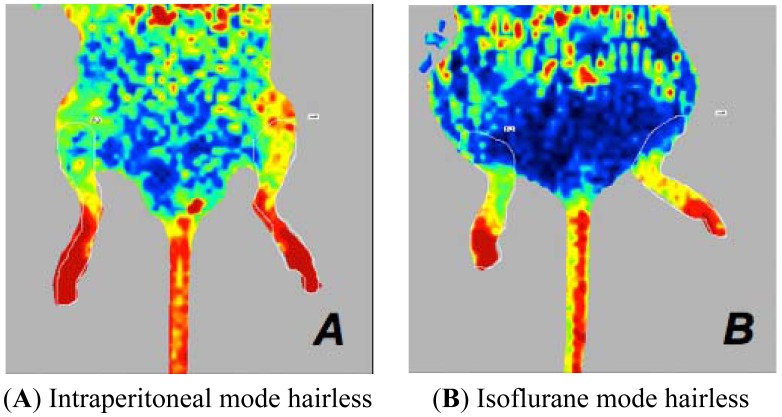
Laser Doppler images of the hind limbs of mice. Perfusion signal is associated with the type of anaesthesia when the mouse is placed in dorsal recumbency. The mice have a body temperature of 38 °C and ARD depilation (A,B). In this experimental condition, mice anaesthetised with intraperitoneal drugs (**A**) have perfusion values higher than mice anaesthetised with isoflurane (**B**).

When the mouse is placed in the dorsal decubitus position, significant differences were associated with the following variables:

1. the mode of depilation in mice anaesthetised with isoflurane at 36 °C or 38 °C. The ARD mice presented perfusion values higher than those of ARC mice (Figure 3).
2. the type of anaesthesia in mice subjected to ARC or ARD. Mice anaesthetised with injectable anaesthesia have higher values of perfusion. (Figures 4 and 5).
3. the body temperature in mice anaesthetised with isoflurane, whether depilated via ARC or ARD. A mouse body temperature of 38 °C yields higher perfusion values. (Figure 3).

**Figure 6.**
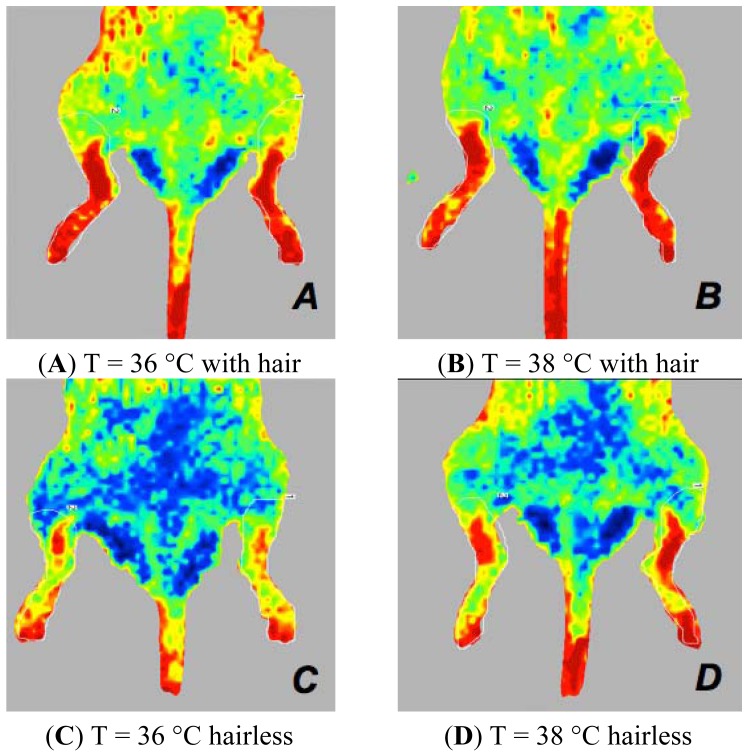
Laser Doppler images of the hind limbs of ARC and ARD mice positioned in the ventral decubitus position. Mice anaesthetised with isoflurane, both at 36 °C and 38 °C, with ARC depilation (**A**,**B**) have values of perfusion higher than those found under similar experimental conditions but with ARD depilation (**C**,**D**).

**Figure 7.**
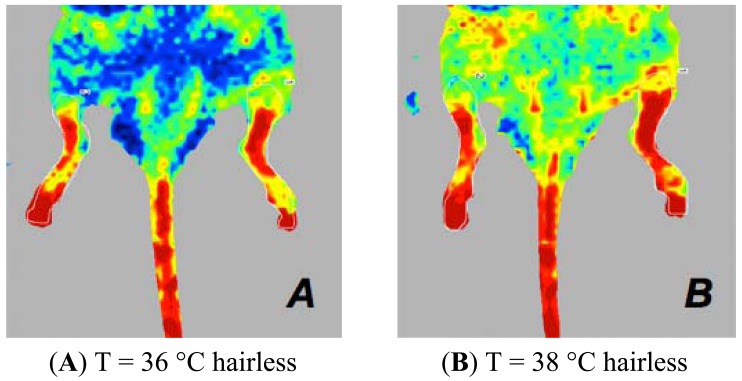

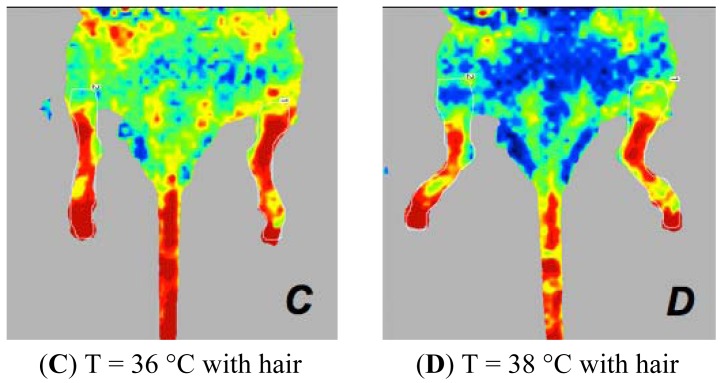
Laser Doppler images of the hind limbs of mice in the presence or absence of hair when the mouse is positioned in ventral decubitus position. Mice anaesthetised with intraperitoneal drugs, both at 36 °C and 38 °C, with ARD depilation (**A**,**B**) have higher perfusion values than those found under similar experimental conditions but with ARC depilation (**C**,**D**).

**Figure 8.**
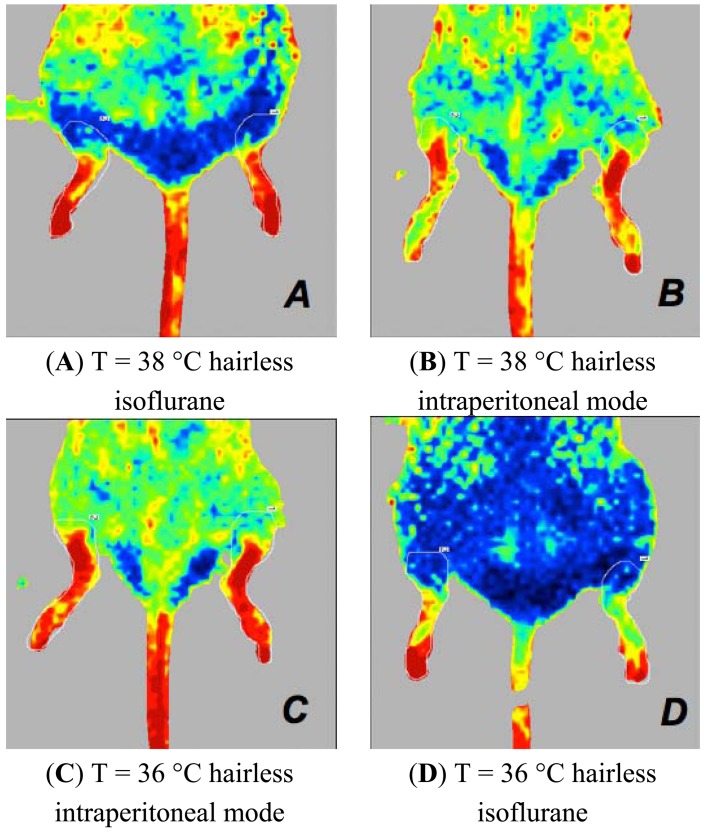
Laser Doppler images of the hind limbs of mice associated with the type of anaesthesia when the mouse is positioned in the ventral decubitus position. Mice anaesthetised with isoflurane have higher perfusion values when depilated with a depilatory cream and have a temperature of 38 °C (**A**) compared to mice that are under similar experimental conditions but are intraperitoneally anesthetised (**B**). Mice anaesthetised with intraperitoneal drugs and depilated with the cream have higher perfusion values at 36 °C (**C**) compared to mice that are under similar experimental conditions but are anaesthetised with isoflurane (**D**).

**Figure 9.**
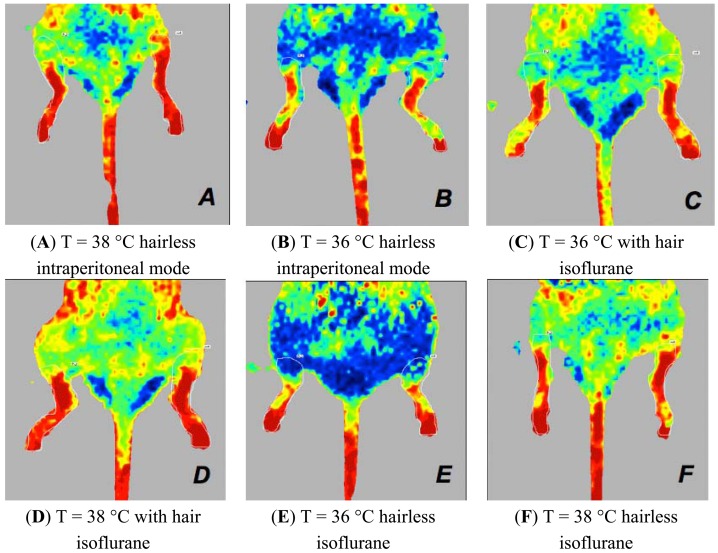
Laser Doppler images of the hind limbs of mice associated with the body temperature when the mouse is positioned in the ventral decubitus position. In mice anaesthetised with intraperitoneal drugs (**A**,**B**), there is a significant difference in both mice. Mice that have a body temperature of 38 °C (A) always have higher perfusion values. In mice anaesthetised with isoflurane (**C**,**D**,**E**,**F**), with ARC (C,D) or ARD (E,F) depilation, and those with a body temperature of 38 °C (D,F) show the highest perfusion values.

When the mouse is placed in the ventral decubitus position, significant differences were associated with the following:

1. Among mice anaesthetised with isoflurane, those depilated with the clipper have higher perfusion values than the mice depilated with the ARC. Among mice anaesthetised with injectable agents, ARC-depilated mice have higher perfusion values than the mice shaved with a clipper (Figures 6 and 7).
2. ARC-depilated mice anaesthetised by injection and with a 36 °C body temperature have higher values. In contrast, mice anaesthetised with isoflurane, with fur and with 38 °C body temperature have higher values (Figure 8).
3. The body temperature in mice anaesthetised with isoflurane, in ARC and ARD mice. There is a significant difference in perfusion values by body temperature in ARC-depilated mice anaesthetised by injection. The perfusion values in mice with a body temperature of 38 °C are always higher (Figure 9).

## 4. Discussion

Our Bland-Altman analysis [13] indicated an excellent correlation between measurements made by the same operator (*i.e*., repeatability) under the same experimental conditions and by two different operators (*i.e*., reproducibility) in measurements made in 20 mice (Figures 1 and 2). Statistically significant differences in perfusion values were observed in different experimental groups. The different experimental conditions (e.g., the presence or absence of hair, the type of anaesthesia, body temperature and position) significantly influence the haemodynamic parameters of mouse microcirculation and the perfusion signal. In particular, the dorsal *versus* ventral body position was the most influential factor. This effect could be related to the anatomy of the cardiovascular system of the hind limb and due to the mouse's respiratory motion. The statistical analysis of measurements collected in the ventral decubitus position reflects a random variation. In this position, the blood vessels of the hind limbs are not oriented toward the laser beam, the skin thickness is greater than that of the skin that is exposed in the dorsal decubitus position and more artefacts are associated with the breathing movements. For these reasons, we considered only statistical data collected in the dorsal decubitus position. In conclusion, we believe that the dorsal decubitus position allows more reliable perfusion data. The presence of hair attenuates the LDPI signal. Mice depilated with a cream show perfusion values higher than those depilated with a clipper. In fact when the mouse is shaved with the razor, the thick undercoat is not removed properly, and the hair absorbs laser light, preventing it from interacting with erythrocytes. Furthermore, the clipper irritates the skin and influences the flow signal. Regarding the temperature, we recommend that mice be maintained at 36 °C during the LDPI exam. Furthermore, the normal mouse body temperature is 36 °C, and there is no need to stress the mice by raising the body temperature.

Anaesthesia-related changes in regional blood flow result from a combination of central circulatory effects on cardiac output and arterial pressure and from local microvascular regulation, with a net result of adjusting the peripheral perfusion to meet the metabolic needs of the tissues [14–16]. The regulatory mechanisms of the microcirculation are mainly via autonomic sympathetic and parasympathetic nerves that reach the vascular smooth muscle. In our study, we tested two anaesthetic protocols (*i.e*., inhalation anaesthesia with isoflurane plus oxygen and injectable anaesthesia with ketamine and xylazine). Several preclinical studies have suggested the use of injectable anaesthesia protocols to perform laser Doppler scanning in mice. Kragh and colleagues [11] used LDPI for non-invasive skin measurements of angiogenic and anti-angiogenic activity in nude mice anaesthetised with ketamine (100 mg/kg) and xylazine (10 mg/kg), whereas Yang and colleagues [17] used the same combination to evaluate skin and hind paw blood perfusion in a mouse model of atherosclerosis. Michauld and colleagues [18] instead used the combination of ketamine (100 mg/kg) and midazolam (5 mg/kg) to assess the blood perfusion in a murine ischemic hind limb model by laser Doppler. Investigators should carefully select an anaesthetic protocol for use during the evaluation of blood perfusion and should carefully consider the relevant effects of different anaesthetics on regional blood flow.

We observe significantly lower perfusion values in mice anaesthetised with inhaled isoflurane plus oxygen *versus* those anaesthetised with injected ketamine plus xylazine. Several preclinical studies indicated that the choice of anaesthetic protocol for studies of blood perfusion is relevant because of the differential effects of anaesthetics on regional blood flow [15]. The type of anaesthesia influences hind limb perfusion values; however, these considerations can fail if attention is not paid to the methods of hair removal in mice. As shown in Figures 7 and 8, perfusion values are influenced by the presence or the absence or hair, and the type of depilation chosen, in addition to the type of anaesthesia.

Inhalation anaesthesia offers the advantages of safe and rapid anaesthesia, and fast, complete recovery that interferes less with the microcirculation [16]. Isoflurane produces slight effects on the haemodynamics of mice, and a dose level of 1.5% yields stable mean arterial pressures and heart rate values comparable to those observed in the animal's conscious state. By contrast, alpha-agonists may interfere with study results because they induce early peripheral vasoconstriction; therefore, ketamine-alpha-agonist combinations are not suitable for studies of vascular smooth muscle. An anaesthetic protocol that provides good immobilisation and relaxation of short duration or with quick reversibility should be used for LDPI. As previously described, because the early peripheral vasoconstriction induced by α-agonists may interfere with study results, ketamine-α-agonist combinations are not suitable for LDPI studies, although ketamine-benzodiazepine combinations can be adequate for immobilisation and do offer the opportunity to reverse one of the agents. In conclusion, Laser Doppler Perfusion Imaging is a useful and minimally invasive method to study mouse hind limb perfusion. This technique is useful for evaluating animal models of ischemia and reperfusion of the hind limbs. However, guidelines for the use of this technique in laboratory mice do not exist. The aim of our work was to optimise the technical parameters and rules for the implementation of the LDPI method. In the future, our findings may not only reduce the number of animals needed for experimentation, but may also help to increase the reliability of the resulting data.

## 5. Conclusions/Outlook

Based on the material presented in this article and within the limits of our experimental conditions, several conclusions were reached. The evaluation of microcirculation with LDPI is of particular interest in clinical and experimental research. There are many factors that may cause significant variations in blood flow detectable by LDPI, such as cardiac activity, temperature, sympathetic activity, anaesthesia, and body positioning. For these reasons, studies performed by laser Doppler should be considered to be a complementary method to others that are already standardised, such as Doppler Ultrasound for the measurement of blood flow. However, compliance with the experimental conditions described in this article will ensure a precise and more reliable method for the study of percutaneous microcirculation.

## Figures and Tables

**Table 1.** Descriptive Statistics. These values are reported as medians and interquartile ranges for the ventral decubitus position.

**Hair**	**Anaesthesia**	**Value**	**Temperature**					

			**36 °C**			**38 °C**		

			25th Percentile	Median	75th Percentile	25th Percentile	Median	75th Percentile

ARD	inhalational	Min	0.69	0.73	0.79	0.72	0.80	0.87
		Median	1.64	1.77	1.91	1.88	2.08	2.25
		Mean	1.78	1.92	2.04	2.04	2.22	2.35
		Max	3.87	4.45	4.89	4.67	5.13	5.81
	intraperitoneal	Min	0.60	0.72	0.80	0.76	0.88	1.02
		Median	1.80	1.92	2.16	1.99	2.19	2.42
		Mean	2.06	2.19	2.37	2.15	2.42	2.60
		Max	5.72	6.36	6.93	5.57	6.07	6.37
ARC	inhalational	Min	0.60	0.77	0.93	0.85	1.06	1.26
		Median	1.72	2.01	2.66	2.13	2.66	3.31
		Mean	1.86	2.19	3.54	2.30	2.79	4.34
		Max	4.02	5.80	10.00	5.03	6.79	10.00
	intraperitoneal	Min	0.35	0.42	0.46	0.40	0.43	0.49
		Median	1.61	1.79	1.99	1.76	1.88	2.01
		Mean	2.01	2.09	2.24	2.00	2.12	2.17
		Max	6.48	7.13	7.93	5.76	6.17	6.81

**Table 2.** Descriptive Statistics. These values are reported as medians and interquartile ranges for the dorsal decubitus position.

**Hair**	**Anaesthesia**	**Value**	**Temperature**					

			**36 °C**			**38 °C**		

			25th Percentile	Median	75th Percentile	25th Percentile	Median	75th Percentile

ARD	inhalational	Min	0.55	0.65	0.72	0.69	0.73	0.85
		Median	1.23	1.38	1.63	1.62	2.18	2.54
		Mean	1.34	1.44	1.74	1.73	2.24	2.59
		Max	2.76	3.16	3.50	3.57	4.13	5.17
	intraperitoneal	Min	0.41	0.59	0.72	0.50	0.64	0.71
		Median	1.75	1.96	2.16	1.97	2.08	2.24
		Mean	1.89	2.25	2.36	2.12	2.26	2.38
		Max	4.90	5.54	6.02	4.79	5.25	5.98
ARC	inhalational	Min	0.34	0.40	0.43	0.39	0.45	0.51
		Median	0.85	0.97	1.08	1.26	1.45	1.61
		Mean	0.88	0.99	1.11	1.44	1.64	1.74
		Max	1.65	1.95	2.48	3.72	4.27	4.73
	intraperitoneal	Min	0.50	0.54	0.61	0.54	0.57	0.65
		Median	1.49	1.83	2.01	1.74	1.98	2.11
		Mean	1.99	2.20	2.38	2.21	2.29	2.37
		Max	5.60	6.04	6.38	5.44	5.82	6.32

**Table 3.** Inferential Statistics. The results of a Mann-Whitney test for the effects of hair, anaesthesia and temperature when the mouse is placed in the dorsal decubitus position.

**Hair**				

Temperature	Anaesthesia	Hair	Mean–Average of the ranks	Median–Average of the ranks
36 °C	inhalational	ARC	14.62	14.96
		ARD	34.33	34.04
			*p* < *0.001*	*p* < *0.001*
38 °C	inhalational	ARC	15.96	15.31
		ARD	33.04	33.69
			*p* < *0.001*	*p* < *0.001*

**Anaesthesia**				

Temperature	Hair	Anaesthesia	Mean–Average of the ranks	Median–Average of the ranks
36 °C	ARD	inhalational	13.88	14.29
		Intraperitoneal	35.13	34.71
			*p* < *0.001*	*p* < *0.001*
36 °C	ARC	Inhalational	12.56	13.58
		Intraperitoneal	36.44	35.42
			*p* < *0.001*	*p* < *0.001*
38 °C	ARC	inhalational	13	16.46
		intraperitoneal	36	32.54
			*p* < *0.001*	*p* < *0.001*

**Temperature**				

Anaesthesia	Hair	Temperature	Mean–Average of the ranks	Median–Average of the ranks
inhalational	ARD	36 °C	15	15.02
		38 °C	34	33.98
			*p* < *0.001*	*p* < *0.001*
Inhalational	ARC	36 °C	14.25	14.98
		38 °C	34.75	34.02
			*p* < *0.001*	*p* < *0.001*

**Table 4.** Inferential Statistics. The results of a Mann-Whitney test for the effects of hair, anaesthesia and temperature when the mouse is placed in the ventral decubitus position.

**Hair**				

Temperature	Anaesthesia	Hair	Mean–Average of the ranks	Median–Average of the ranks
36 °C	inhalational	ARC	28.9	28.79
		ARD	20.1	20.21
			*p* < *0.001*	*p* < *0.001*
36 °C	intraperitoneal	ARC	20.46	20.02
		ARD	28.54	28.94
			*p* < *0.001*	*p* < *0.001*
38 °C	inhalational	ARC	30.85	30.75
		ARD	18.15	18.25
			*p* < *0.001*	*p* < *0.001*
38 °C	intraperitoneal	ARC	*17.31*	*17.02*
		ARD	31.69	31.98
			*p* < *0.001*	*p* < *0.001*

**Anaesthesia**				

Temperature	Hair	Anaesthesia	Mean–Average of the ranks	Median–Average of the ranks
36 °C	ARD	inhalational	15.48	19.02
		intraperitoneal	33.52	29.98
			*p* < *0.001*	*p* < *0.001*
38 °C	ARC	inhalational	32.73	32.6
		intraperitoneal	16.27	16.4
			*p* < *0.001*	*p* < *0.001*

**Temperature**				

Anaesthesia	Hair	Temperature	Mean–Average of the ranks	Median–Average of the ranks
inhalational	ARD	36 °C	15.69	16.15
		38 °C	33.31	32.85
			*p* < *0.001*	*p* < *0.001*
intraperitoneal	ARD	36 °C	20.06	18.65
		38 °C	28.94	30.35
			*p* < *0.001*	*p* < *0.001*
inhalational	ARC	36 °C	20.73	19.29
		38 °C	28.27	29.71
			*p* < *0.001*	*p* < *0.001*

## References

[1] Paigen K. (2002). Understanding the human condition: Experimental strategies in mammalian genetics. ILAR J..

[2] Condeelis J., Weissleder R. (2010). *In vivo* imaging in cancer. Cold Spring Harb. Perspect. Biol..

[3] Massoud T.F., Gambhir S.S. (2003). Molecular imaging in living subjects: Seeing fundamental biological processes in a new light. Genes Dev..

[4] Sarnik S., Hofirek I., Sochor O. (2007). Laser Doppler fluxmetry. Biomed. Pap. Med. Fac. Univ. Palacky Olomouc Czech Repub..

[5] Laser Doppler Flowmetry-A Theoretical Framework. http://www.imt.liu.se/bit/ldf/ldfmain.html.

[6] Key H., Jackson P.C., Wells P.N. (1988). New approaches to transillumination imaging. J. Biomed. Eng..

[7] Humeau A., Steenbergen W., Nilsson H., Strömberg T. (2007). Laser Doppler perfusion monitoring and imaging: Novel approaches. Med. Biol. Eng. Comput..

[8] Niiyama H., Huang N.F., Rollins M.D., Cooke J.P. (2009). Murine model of hindlimb ischemia. J. Vis. Exp..

[9] Rayssac A., Neveu C., Pucelle M., van den Berghe L., Prado-Lourenco L., Arnal J.F., Chaufour X., Prats A.C. (2009). IRES-based vector coexpressing FGF2 and Cyr61 provides synergistic and safe therapeutics of lower limb ischemia. Mol. Ther..

[10] Cho W.G., Albuquerque R.J., Kleinman M.E., Tarallo V., Greco A., Nozaki M., Green M.G., Baffi J.Z., Ambati B.K., De Falco M. (2009). Small interfering RNA-induced TLR3 activation inhibits blood and lymphatic vessel growth. PNAS.

[11] Kragh M., Quistorff B., Kristjansen P.E.G. (2001). Quantitative estimates of angiogenic and anti-angiogenic activity by laser Doppler flowmetry (LDF) and near infra-red spectroscopy (NIRS). Eur. J. Cancer.

[12] (2011). Committee for the Update of the Guide for the Care and Use of Laboratory Animals; National Research Council. Guide for the Care and Use of Laboratory Animals.

[13] Bland J.M., Altman D.G. (1986). Statistical methods for assessing agreement between two methods of clinical measurement. Lancet.

[14] Yang X.P., Liu Y.H., Rhaleb N.E., Kurihara N., Kim H.E., Carrettero O. (1999). Echocardiographic assessment of cardiac function in conscious and anesthetized mice. Am. J. Physiol. Heart Circ. Physiol..

[15] Constantinides C., Mean R., Janssen B.J. (2011). Effects of isoflurane anesthesia on the cardiovascular function of the C57BL/6 mouse. ILAR J..

[16] Gargiulo S., Greco A., Gramanzini M., Esposito S., Affuso A., Brunetti A., Vesce G. (2012). Mice anesthesia, analgesia, and care, part II: Special considerations for preclinical imaging studies. ILAR J..

[17] Yang X.P., Liu Y.H., Rhaleb N.E., Kurihara N., Kim H.E., Carrettero O. (1999). Echocardiographic assessment of cardiac function in conscious and anesthetized mice. Am. J. Physiol. Heart Circ. Physiol..

[18] Michauld S.E., Menard C., Guy L.G., Gennaro G., Rivard A. (2003). Inhibition of hypoxia-induced angiogenesis by cigarette smoke exposure: Impairment of the HIF-1alpha/VEGF pathway. FASEB J..

